# Experimental Metabolic Syndrome Model Associated with Mechanical and Structural Degenerative Changes of the Aortic Valve

**DOI:** 10.1038/s41598-018-36388-y

**Published:** 2018-12-13

**Authors:** Jason L. Go, Komal Prem, Mohammed A. Al-Hijji, Qing Qin, Christopher Noble, Melissa D. Young, Lilach O. Lerman, Amir Lerman

**Affiliations:** 1 Department of Cardiovascular Medicine, Mayo Clinic, 200 First Street SW, Rochester, 55905 MN USA; 2 Department of Molecular Pharmacology and Experimental Therapeutics, Mayo Clinic, 200 First Street SW, Rochester, 55905 MN USA; 3 Department of Nephrology and Hypertension, Mayo Clinic, 200 First Street SW, Rochester, 55905 MN USA

## Abstract

The purpose of this study was to test the hypothesis that an experimental high fat (HF) animal with metabolic syndrome results in structural degeneration of the aortic valve. Domestic pigs were divided (n = 12) and administered either a normal or HF diet. After 16-weeks, the HF diet group had increased weight (p ≤ 0.05), total cholesterol (p ≤ 0.05), and systolic and diastolic pressure (p ≤ 0.05). The aortic valve extracellular matrix showed loss of elastin fibers and increased collagen deposition in the HF diet group. Collagen was quantified with ELISA, which showed an increased concentration of collagen types 1 and 3 (p ≤ 0.05). In the HF diet group, the initial stages of microcalcification were observed. Uniaxial mechanical testing of aortic cusps revealed that the HF diet group expressed a decrease in ultimate tensile strength and elastic modulus compared to the control diet group (p ≤ 0.05). Western blot and immunohistochemistry indicated the presence of proteins: lipoprotein-associated phospholipase A2, osteopontin, and osteocalcin with an increased expression in the HF diet group. The current study demonstrates that experimental metabolic syndrome induced by a 16-week HF diet was associated with a statistically significant alteration to the physical architecture of the aortic valve.

## Introduction

Aortic valve disease is the third most common cardiovascular disorder in the United States with valve calcification and degeneration being the most common pathology^1–4^. The aortic valve enters a process of sclerosis where it becomes mildly thickened and calcific, but initially does not cause obstruction. Continued thickening results in fibrocalcific remodeling that causes severe calcification and impairs valvular motion^5^. Functionality and durability of the valve can be evaluated by measuring the tensile strength and elastic properties of the cusps^6,7^. Valve strength and elasticity are dependent on the concentration of collagen, elastin, and proteoglycans found within the scaffold^6,8,9^.

Metabolic syndrome (MetS) is associated with comorbidities that contribute to overall cardiovascular health. Rodents are commonly used in cardiovascular research, but these models do not capture the vascular anatomy, physiology, or pathology found in humans. A MetS model that is widely utilized for its ability to elucidate the pathophysiology of MetS in humans is domestic pigs^10–12^. Zhang *et al*. has described this model in greater depth and observed that MetS-induced pigs develop increased abdominal circumference and concentrations of serum insulin and total cholesterol compared to male pigs making it an appropriate model in studying the effects of MetS^10–12^. Studies have postulated that the metabolic stress associated with a high fat (HF) diet leads to the pathogenesis of calcific aortic stenosis and atherosclerosis^13^. Cardiovascular risk factors such as MetS and diabetes have been shown to predict future events of valvular heart disease (VHD) and aortic calcification over time^4^.

Although the signaling pathways in VHD have yet to be identified, three major pathophysiological processes have been implicated in the development of aortic calcification and degerenation^1^. The first process involves the action of osteoblast-like cells that promote calcification similar to bone formation and blood vessel calcification after endothelial injury^2^. The second process involves the oxidation of low-density lipoproteins (oxLDL) and phospholipids by lipoprotein-associated phospholipase A_2_ (Lp-PLA_2_), forming free fatty acids (FFA) and lysophosphotidylcholine (LPC)^3^. Recently, studies have investigated the potential role of oxLDL and Lp-PLA_2_ in aortic valve (AV) degeneration^3,5,14,15^. The final process involves valvular endothelial cells (VECs) that communicate with valvular interstitial cells (VICs) to initiate extracellular remodeling. Unlike atherosclerotic disease, there are no known preventive measures for the progression of AV calcification and degeneration where the current treatment is aortic valve replacement (AVR)^5^. The current study was designed to test the hypothesis that a clinically relevant experimental animal model given a HF diet will lead to the physical and structural changes of the aortic valve.

## Methods

### Ethical Statement

American Yorkshire domestic pigs were housed together at the Mayo Clinic Institutional Hills Farm and treated according to the Guide for the Care and Use of Laboratory Animals (National Institute of Health, USA). Standard of care was approved by Mayo Clinic’s Institutional Animal Care and Use Committee (Protocol # A65014) throughout the study.

### Animal Care, Sample Preparation, and *Ex vivo* Tissue Analysis

Pigs (n = 12) were nursed for 28 days and weened into a normal diet for 4 weeks before being transported to the Mayo Clinic facilities. After the initial 7 weeks, 6 pigs were randomly selected and given a HF diet while the remaining 6 pigs continued a normal feeding regimen for 16 weeks^10,12,16,17^. Animals in the control group (n = 6; female) were fed a normal diet consisting of a standard swine feed containing 14.5% protein and 3% fat with 3.3 Kcal/g of feed (Purina Animal Nutrition LLC, Shoreview MN). Animals in the treatment HF group (n = 6; female) were fed a high-fat and high-fructose feed containing 17% protein and 20% fat with 4.1 Kcal/g of feed (TestDiet, St. Louis MO). Additionally, all 12 pigs were implanted with a DSI telemetry system L11 implant (Data Sciences International, New Brighton, MN). Telemetry was surgically implanted 6-weeks into the normal and HF diet treatment and recorded temperature, heart rate, and blood pressure for 10 weeks. At approximately 23–24 weeks of age, blood samples were collected, pigs were euthanized with 100 mg/kg IV pentobarbital, and the aortic valve (including the root and cusps) was excised.

### Radiologic Imaging: Microscopic Computerized Tomography (CT) Scan

One aortic cusp from each group (n = 4) was evaluated using a custom made micro CT scanner (North Star Imaging Company, Rogers, MN) to determine the degree of aortic cusp microcalcification observed in the HF versus the normal diet groups. The scanner has a fixed micro-focus X-ray source (Hamamatsu Photonics K.K, Hamamatsu City, Japan) and a flat panel X-ray detector (Varian Medical Systems, Palo Alto, CA). The scanner includes flexible geometry with spatial resolution ranging from 5–127 um at different focus modes (small, medium, large) and power levels ranging from 40 kVp–150 kVp^12,17,18^.

### Histology and Immunohistochemistry

One aortic cusp from each group (n = 10) was removed, sectioned, and paraffin-embedded. Antibodies were used for immunohistochemical (IHC) staining to express calcification, subendothelial lipid accumulation, and presence of interstitial-like cells in the explanted tissue. Osteopontin (OPN, 1:100) (Abcam, Cambridge, UK; ab8448) and osteocalcin (OCN, 1:100) (Santa Cruz Biotechnology, Dallas, TX; sc-365797) stained for calcification; platelet activating factor acetylhydrolase (PAFAH, 1:150) (Abcam, ab169836) stained for subendothelial lipid accumulation; and α-Smooth Muscle Actin (α-SMA, 1:100) (Abcam, ab5694) was utilized for detecting interstitial-like cell phenotypes. Immunohistochemistry methodology was used in our previous work^6^. Cellularity, DNA, calcification, collagen deposition, and fibrosis were observed using Hematoxylin-Eosin (H&E), Von Kossa, Alizarin red, Masson Trichrome, Movat’s Pentachrome, and Elastin stains, respectively. Images were quantitatively analyzed in terms of expressed surface area using ImageJ software^19^.

### Scanning Electron Microscopy (SEM) and Transmission Electron Microscopy (TEM)

One aortic cusp from each group (n = 4) was fixed in 1% osmium tetroxide (OsO4), dehydrated through a graded series of ethanol, and embedded in Spur resin. Samples were used to determine the aortic cusp collagen orientation from each group. Sections were cut with an ultra-microtome (100 nm or 0.1 μm), stained in 3% (w/v) uranyl acetate in 70% (v/v) EtOH for 20 min, and then stained in Reynold’s lead citrate for 20 min. Imaging was captured using the JEOL “JEM-1400 Plus” transmission electron microscope (JEOL USA, Inc. Peabody, MA)^20^.

### Western Blots

Aortic cusp (1/2 of 1 cusp per animal from each group; n = 8) samples were homogenized and proteins extracted in RIPA lysis buffer (Thermo Fischer, Waltham, MA). Protein concentration of each sample was assessed through a BioRad Protein Reagent (BioRad Laboratories, Hercules, CA) and read utilizing a spectrophotometer. Concentration was confirmed with the Nanodrop One (Thermo Fisher Scientific, Waltham, MA) and ranged from 2 µg/µL–3 µg/µL. Membranes were incubated with primary antibody at 4 °C overnight while rocking (anti-OPN polyclonal [1:1000, Abcam ab8448], anti-OCN monoclonal [1:1000, Santa Cruz sc-365797], and anti-PAFAH polyclonal [1:1000, Abcam ab169836]). Immunodetection was performed with the SuperSignal^TM^ West Pico PLUS Chemiluminescent Substrate (Thermo Fischer, Waltham, MA) and imaged on the UVP imager using VisionWorks 8.2 (Analytik-Jena US, Inc., Beverly, MA). Membrane images were analyzed using ImageJ software^19^.

### ELISA Quantification of Collagen Content

Aortic cusp (1/2 of 1 cusp per animal from each group; n = 12) was used. Collagen type 1(COL1A1 ELISA kit Porcine Collagen Type I, alpha 1 (CO1A1) ELISA kit-AAH36531.1) and type 3 (COL3 ELISA kit Porcine Collagen Type III (COL3) ELISA kit-EAX10911.1) were quantified via a 96-Strip well sandwich ELISA kit. Samples were prepared according to the directions outlined per the kit manufacturer’s suggestion (MyBioSource, Inc. San Diego, CA). Intra-assay and inter-assay coefficients of variation values were <10% and <12%, respectively. The lower limit of detection of collagen types 1 and 3 was 1.56–100 ng/mL. All tests for each sample were performed in duplicate.

### Biomechanical Properties of Porcine Aortic Cusps: Uniaxial Tensile Testing

One cusp (n = 10) from each aortic valve was stored in cool PBS for no more than 30 minutes before processing. The ultimate tensile strength and elastic modulus were measured to determine the mechanical properties of aortic cusps from each group. Cusps were cut into rectangular 12 mm (height) and 4 mm (width) shapes. Collagen fibers were aligned circumferentially and digital calipers were used to measure the thickness at the center. Three thickness measurements were taken, and the average was used for data analysis^6,21^. Two rectangular paper window frames (20 mm × 9 mm) with a rectangular window (12 mm × 5 mm) at the center was used to hold and prevent unwanted damage while mounted into the Instron 5965 tensile tester (Instron Corporation, Norwood MA)^20^. Testing was performed at crosshead displacement speed of 0.1667 mm/s per ASTM standards (ASTM F2150–13)^22^. The ultimate tensile strength and elastic modulus were calculated from the resulting stress-strain graph using load versus displacement data from the Bluehill 3 program (Instron Corporation, Norwood MA).

### Statistical analysis

Continuous variables are presented as mean ± SD or median (25th, 75th percentiles) depending on the normality of distribution. Given the low sample size (N < 25) and non-normal distribution of studied continuous variables, the Mann-Whitney U test was used to analyze HF diet and control diet differences in ultimate tensile strength and elastic modulus properties, histology and immunohistochemistry as percentage of positive signal area visualized under 20x magnification, and Western blot and ELISA as protein concentration visualized with film and plate reading. Statistical significance was defined as a 2-tailed *P* value ≤ 0.05. Statistical analyses were completed utilizing JMP® Pro 10.0.0 (SAS Institute Inc, Cary, North Carolina).

## Results

### *Ex vivo* Macroscopic Tissue Analysis

**Figure 1:**
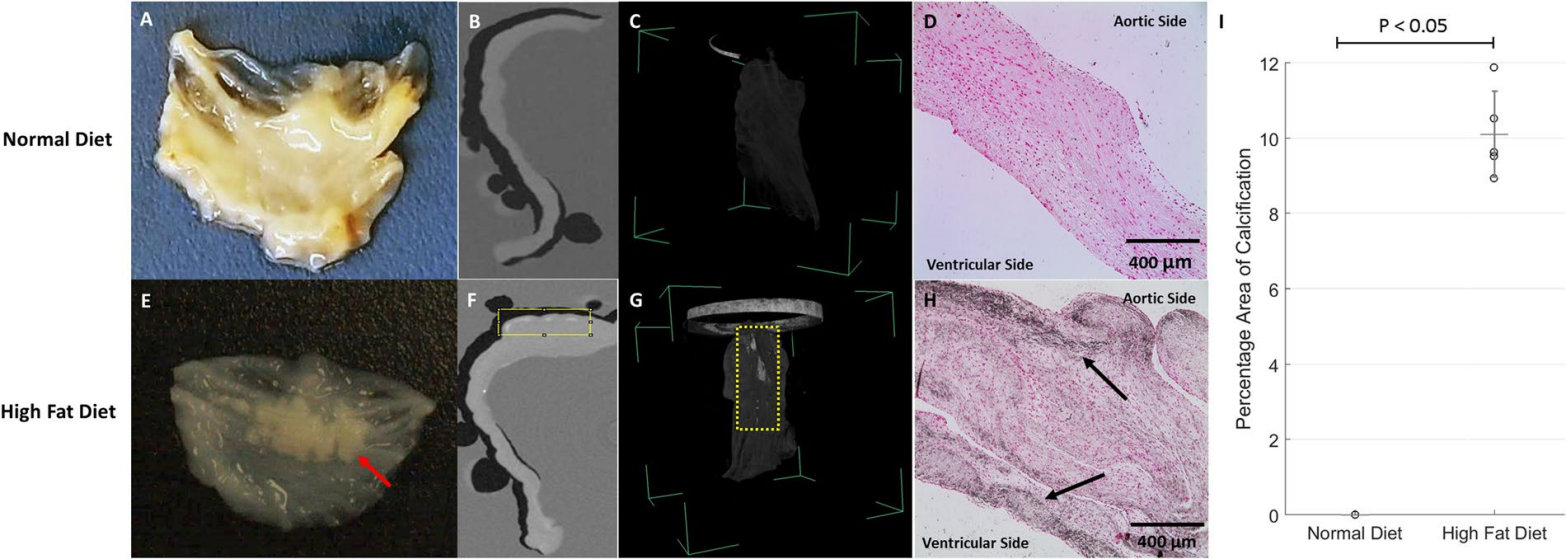
Gross examination (n = 12), Micro Computerized Tomography (CT) Scan (n = 4), and Von Kossa Stain (n = 10) of Metabolic Syndrome Pig Aortic Cusps after 16 Weeks. Normal diet cusp showing no calcification via (**A**) gross examination, (**B**) sliced 2D cross-sectional view, (**C**) 3D volume rendering, and **D**) no evidence of calcium deposit utilizing Von Kossa. High fat diet cusp showing (**E**) Deposited calcific nodules observed (red arrow), (**F**) sliced 2D cross-sectional view showing areas of opacity (yellow box) along the cusp periphery, (**G**) 3D volume rendering showing opacity, and **H**) darken areas (denoted with arrows) shows calcium deposits that are specific to the cusp periphery. (**I**) The percentage area of calcification in the HF diet group statistical significance (P < 0.05).

After a 16-week period, there was no inflammation on the valve cusps from either group (n = 6 HF diet and n = 6 normal diet pigs). Vital signs, weight, blood pressure, and heart rate were routinely recorded via telemetry, which showed an increase in all areas for the HF diet group at sacrifice compared to 6 weeks into arrival. Blood draw obtained at sacrifice measured glucose and cholesterol levels and showed an increase in the HF diet group (Table 1). Additionally, gross calcific nodules were present on the cusps of the HF diet group (Fig. 1E).

**Table 1:** Average Animal Weight, Blood pressure, Heart Rate, and Lipid Profile After 16 Weeks of Diet (Mean and Standard Deviation).

	Control Diet	High Fat Diet
Weight (kg)	64.7 ± 2	87.3 ± 5*
Systolic (mmHg)	115.4 ± 11	129.4 ± 4*
Diastolic (mmHg)	82.2 ± 9	99 ± 9*
Heart Rate (BPM)	102.8 ± 9	78.8 ± 5*
Low Density Lipoproteins [LDL] (mg/dL)	31 ± 8	307 ± 53*
High Density Lipoproteins [HDL] (mg/dL)	45 ± 4	136 ± 25*
Triglycerides (mg/dL)	7 ± 2	12 ± 7
Total Cholesterol (mg/dL)	77 ± 5	446 ± 32*
Glucose (mg/dL)	143.8 ± 44	153.5 ± 44

^*^Statistically significant (P < 0.05).

### Radiologic Imaging: Microscopic Computerized Tomography (CT) Results

There was no evidence of microcalcification found in the control group; however, opaque areas signifying microcalcification were observed from the HF diet group in both 2D and 3D imaging (Fig. 1G). The mean percentage of total opaque surface area in HF diet is 10.7% ± 1.7 and control diet is 0% (p < 0.05).

### Scanning and Transmission Electron Microscopy (SEM/TEM)

**Figure 2:**
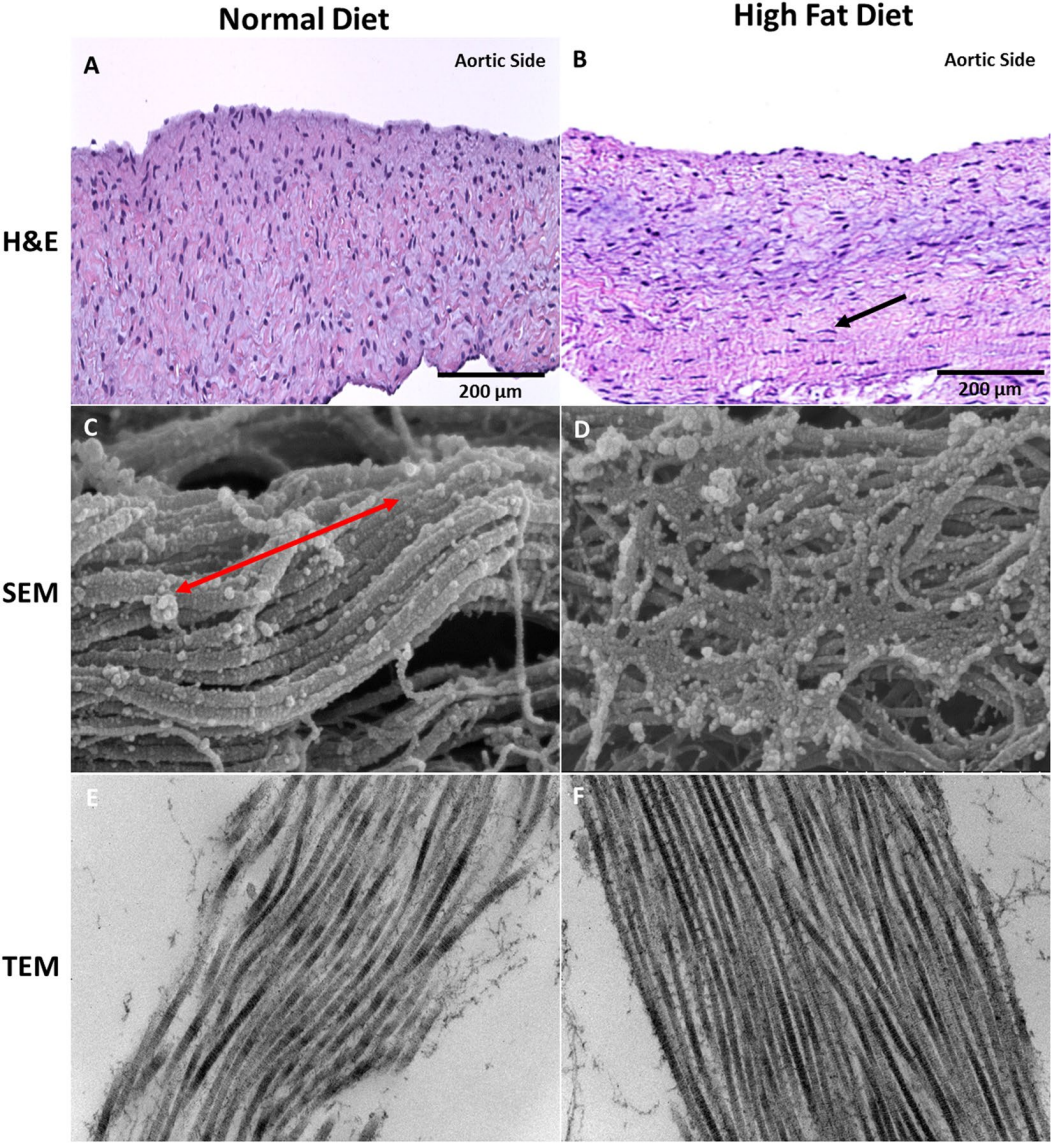
Hematoxylin and Eosin (n = 10) and Scanning and Transmission Electron Microscopy (n = 4) showing the Extracellular Matrix, Inflammatory Cells, and Collagen Architecture After 16 Weeks. (**A**) Aortic cusp from normal diet pig showing the normal ECM without evidence of inflammatory cells (200 µm). (**B**) Aortic cusp from HF diet pig showing elongated inflammatory cells (denoted with arrow) within the cusp between the spongiosa and ventricularis layers (200 µm). (**C**) Uniform collagenous structure (denoted with double-ended red arrow) of the aortic cusp in the circumferential direction in the normal diet group (SEM 500 nm). (**D**) Misaligned collagen structure observed in the high fat diet group aortic cusp (SEM 500 nm). (**E**) TEM Longitudinal view showing collagen fibers in the normal diet group (TEM 200 nm). (**F**) TEM Longitudinal view showing increased collagen fibers in the high fat diet group (TEM 200 nm).

Collagen is aligned in the circumferential direction within the ECM. In the normal diet group, cusps imaged with SEM showed directional uniformity of collagen fibers while cusps from the HF diet were misaligned (Fig. 2D). The same cusps imaged with TEM showed the longitudinal view of collagen fibers in the normal diet group and an increase of the same collagen fibers in the HF diet group (Fig. 2F).

### Analysis of Biomarkers and Quantification of Collagen and Elastin Content

On H&E staining, Fig. 2 shows that the extracellular matrix (ECM) was uniformly arranged in both normal and HF diet groups; however, a small number of inflammatory cells were observed in the HF diet group.

#### Collagen and Elastin

**Figure 3:**
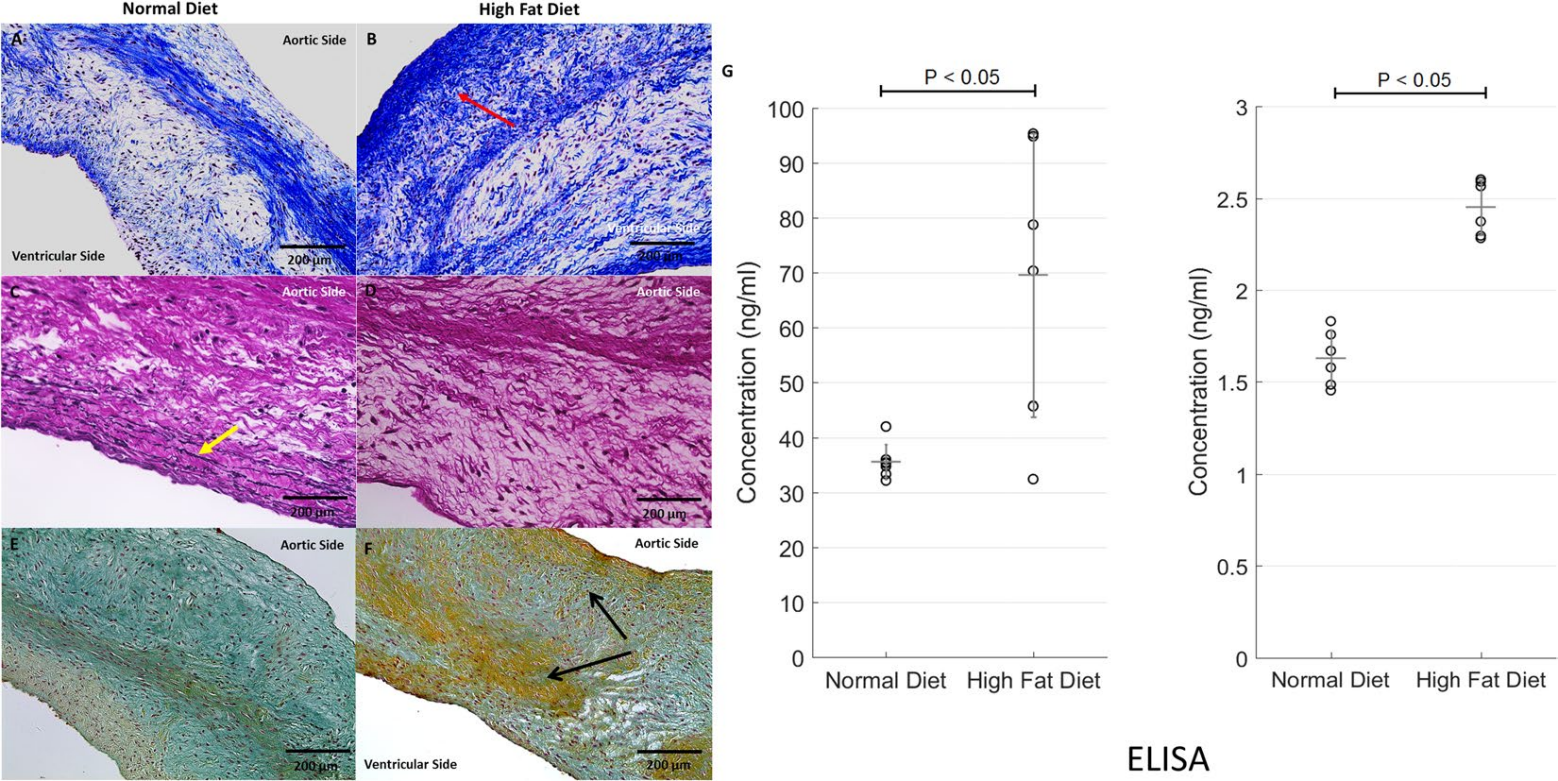
Masson’s Trichrome, Elastin, and Movat’s Pentachrome Histology Distinguishing Collagen and Elastic Fibers in Normal Diet Versus High-fat Diet Pigs After 16 weeks (n = 10). (**A**) Masson’s trichrome: Collagen components observed in normal diet pigs showing the collagenous architecture. (**B**) Increased collagen concentration observed in the HF diet pigs at the fibrosa layer (denoted in red arrow) of the aortic cusps. The collagen threshold area is two-times greater in the HF diet group compared to the normal diet group. This was measured semi-quantitatively by percentage area of collagen on Masson’s Trichrome. (**C**) Elastin fibers (denoted with yellow arrow) found within the normal diet group aortic cusp ECM. (**D**) There were no observable elastin fibers present in the high fat diet group due to the pathological collagen remodeling. (**E**) Movat’s Pentachrome: Normal Diet cusp show uniform ECM architecture containing ground substance and mucins. (**F**) Pigs administered a HF diet showed an increase of collagen within the ECM (denoted with black arrows) in the fibrosa and spongiosa layers of the aortic cusps. (**G**) Quantitative Analysis of Collagen Types 1 and 3 Utilizing ELISA in Normal Diet Versus High Fat Diet Pigs After 16 Weeks (n = 12). Collagen type 1 concentration is increased in HF diet compared to the normal diet group (P < 0.01). Collagen type 3 concentration is increased in HF diet compared to the normal diet group (P < 0.01).

Masson trichrome showed the collagen structure, muscle fiber, and nuclei in cusps from both groups (Fig. 3). There was increased collagen deposition within the fibrosa layer in the HF diet group compared to the control group (Table 2). As expected, elastin fibers were present in cusps from the control group; however, there was loss of elastin in cusps from the HF diet group (Fig. 3D). Collagen quantification utilizing ELISA was performed and showed a statistically significant increase in the concentration of collagen types 1 and 3 in the HF diet compared to the normal diet group. In Fig. 3E, a statistical significance was observed for collagen type 1 (P < 0.05) and collagen type 3 (P < 0.05). Movat’s pentachrome showed similar results in that there was an increase of collagen found within the fibrosa and spongiosa layers in the HF diet group, while only ground substances and mucin were observed in the normal diet group (Fig. 3E,F).

**Table 2:** Average Collagen and Elastin Content Reported in Percentage Area at Explant.

Area	Control Diet	High Fat Diet
Collagen	43.7% ± 18.9	82.9% ± 17.1*
Elastin	28.0.1% ± 2.4	0.0%*

^*^Statistically significant (P < 0.01).

#### Calcium

**Figure 4:**
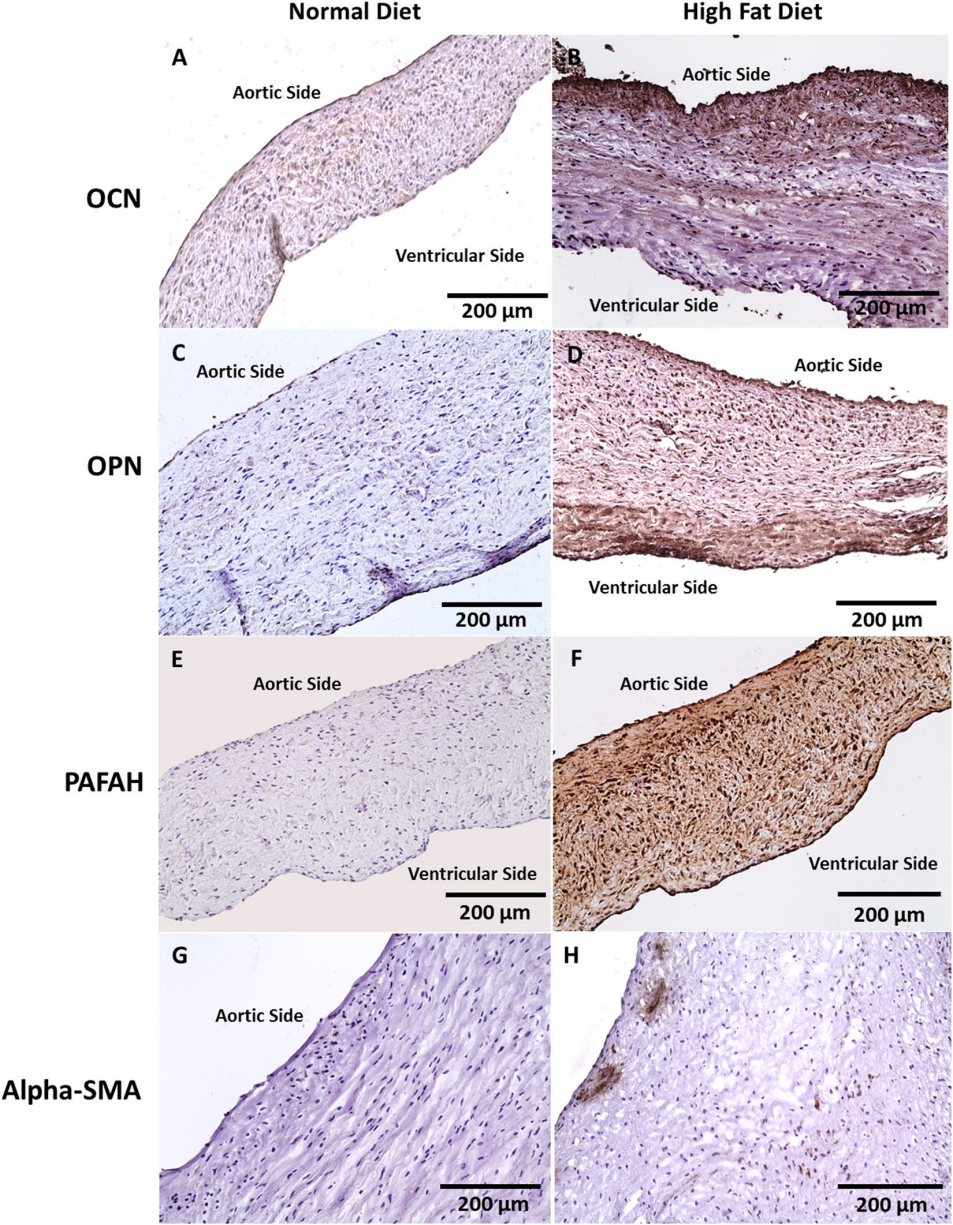
Immunohistochemical Characterization of Aortic Cusps in Metabolic Syndrome Pigs After 16 Weeks (n = 10). (**A,B**) No OCN expression in normal diet cusps. Minimal expression of OCN observed along the endothelium and fibrosa layer (200 µm). (**C,D**) No OPN expression in normal diet cusps. Increased expression of OPN along the endothelium (200 µm). (**E,F**) No PAFAH expression in normal diet cusps. PAFAH within all three layers of the aortic cusp (200 µm). (**G,H**) No Alpha-SMA expression in normal diet cusps. Alpha-SMA expression was observed to be interspersed along the fibrosa of the aortic cusp (200 µm).

**Figure 5:**
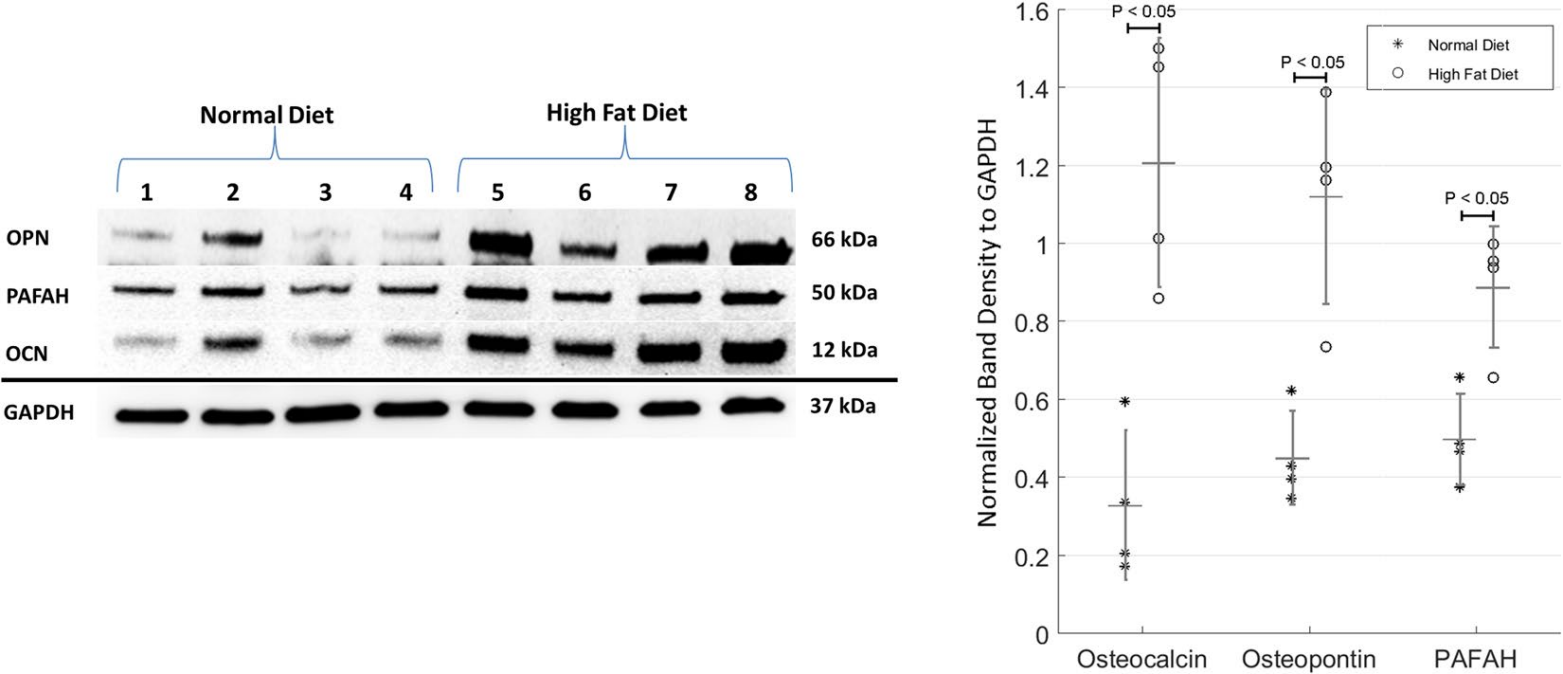
Western Blots Showing Concentration of Various Biomarkers in the Normal Diet Verses High Fat Diet Pigs After 16 Weeks (n = 8). OPN, PAFAH, and OCN expression was present in both the normal diet and high fat diet groups; however, there was a statistically significant increase in the normalized band density to GAPDH of OPN, PAFAH, and OCN in the HF diet group (P < 0.05)

Von Kossa stains showed dark areas of microcalcification (Fig. 1H). On IHC analysis, there was an increased expression of OCN, OPN, and PAFAH in cusps from the HF diet group (Fig. 4). In addition, α-SMA was utilized to assess the cellularity of the cusps and showed myofibroblast-like cell infiltration in the HF diet group (Fig. 4H). Western blot protein analysis was performed with aortic cusp homogenates from each group. Both normal and HF diet cusps showed expression of OCN, OPN, and PAFAH; however, there was a statistically significant increase in the normalized band density to GAPDH of all biomarkers in the HF diet group (P < 0.05) (Fig. 5).

### Biomechanical Properties of Porcine Aortic Valves: Uniaxial Tensile Testing

**Figure 6:**
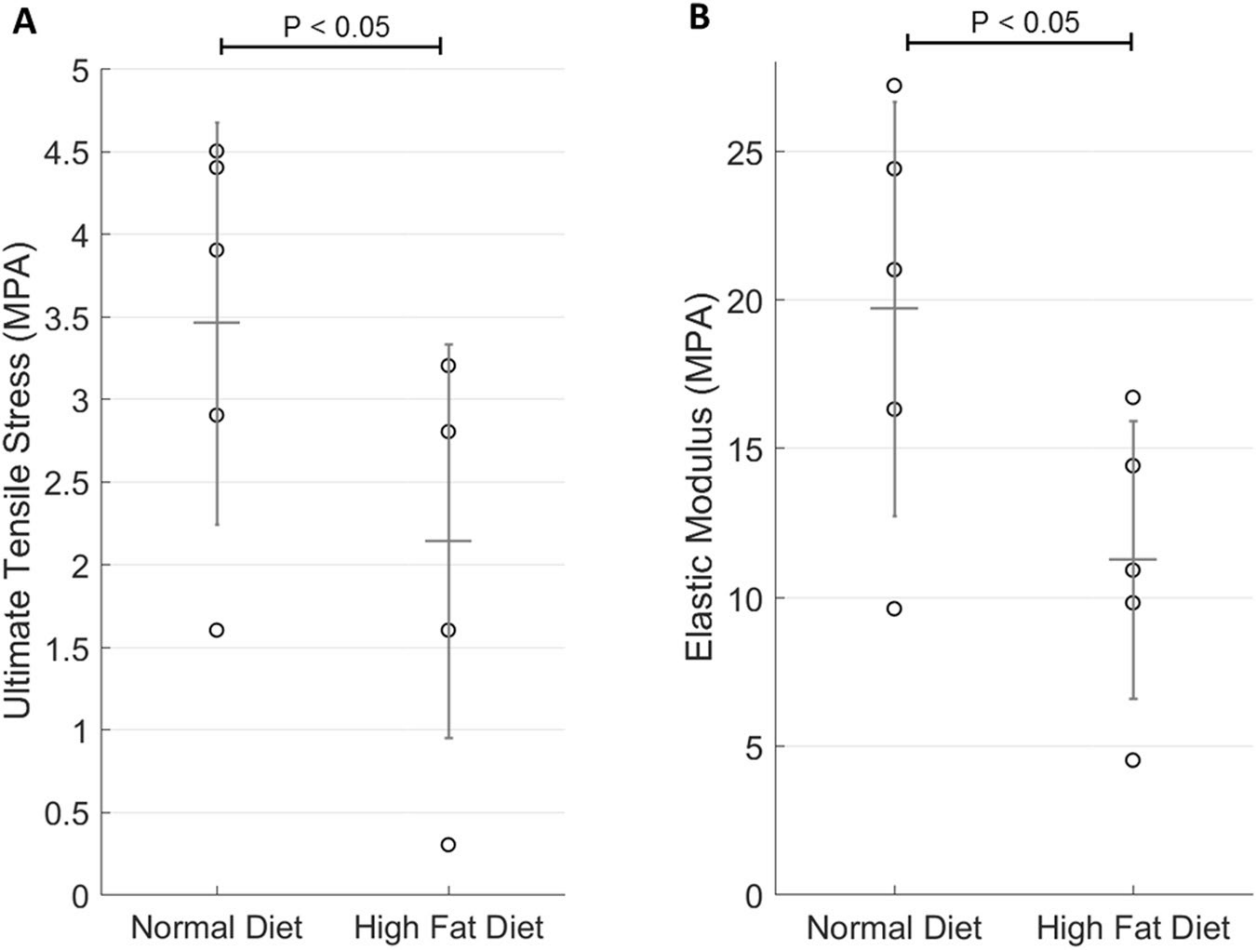
Mechanical Properties of Aortic Cusps in Normal Diet Versus High Fat Diet Pigs After 16 Weeks (n = 10). (**A**) The ultimate tensile stress in high fat diet group was significantly decreased compared to the normal diet group control (2.60 ± 0.71 MPa HF diet group vs 3.92 ± 0.72 MPa control diet group; P < 0.05). (**B**) The stress strain curves demonstrated a statistically significant decrease in elastic modulus in the HF diet group (13.7 ± 2.60 MPa HF diet group vs. 22.9 ± 5.74 MPa control diet group; P < 0.05).

As seen in Fig. 6, specimens were tested in the circumferential direction (n = 10). The stress-strain curves demonstrated a statistically significant decrease in elastic modulus in the HF diet group. There was also a statistically significant difference observed in the ultimate tensile strength when comparing the HF diet group to the control. The mean and standard deviation for cusp thickness was 0.56 mm ± 0.20 for the control group and 0.82 mm ± 0.30 for the HF diet group (p < 0.05).

## Discussion

**Figure 7:**
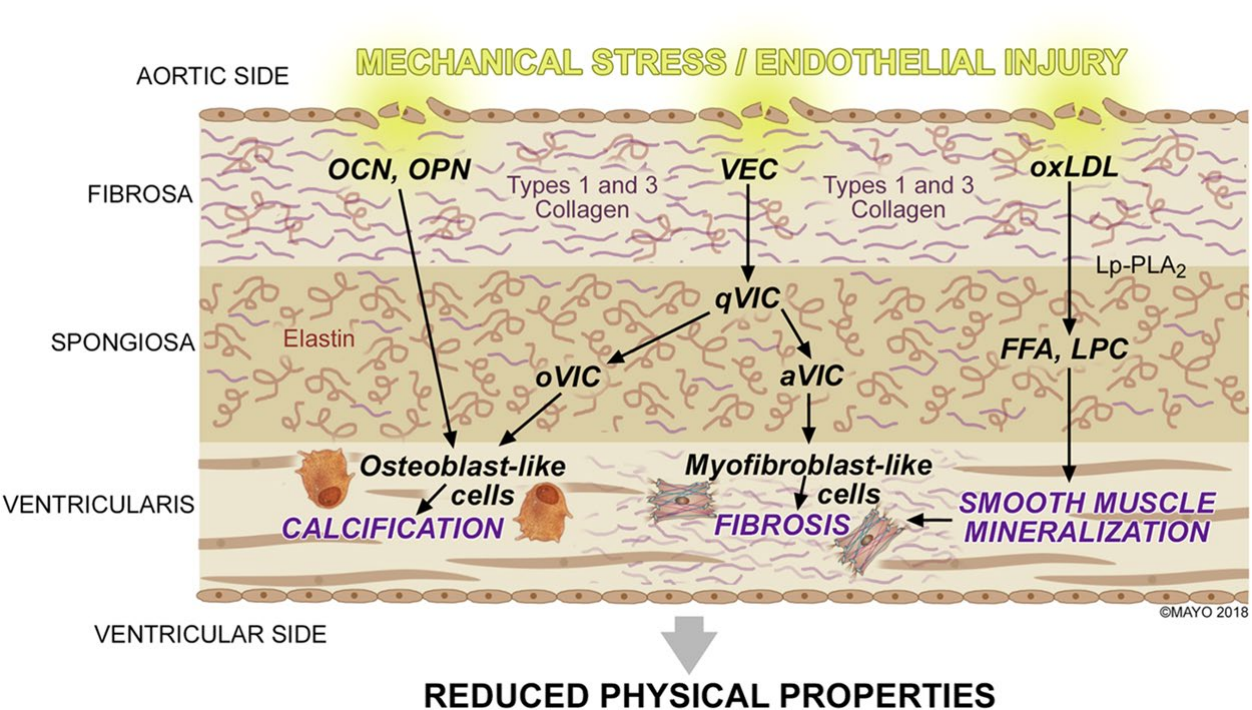
Summary of the Pathophysiology within the Aortic Valve Cusp During Calcification and Degeneration.

The study demonstrated that experimental metabolic syndrome (MetS) in pigs induced by a high fat (HF) diet for 16 weeks resulted in mechanical and structural aortic valve degeneration and calcification. These changes were characterized by a statistically significant decrease in biomechanical properties. The current study suggests that cardiovascular risk factors that are associated with atherosclerosis may have also contributed to the progression of aortic valve degeneration and calcification (Fig. 7).

Pigs have become widely used in cardiovascular research for similarities to human anatomy and physiology^23^. Studies have utilized pigs with MetS to study the effects of HF diet on aortic calcification^10^. The aortic cusp is composed of a fibrosa, spongiosa, and ventricularis layer that are primarily composed of proteoglycans and the fibrous proteins collagen and elastin. Together, these components are responsible for the biomechanical support and structure of the aortic valve. Other fibrous proteins such as fibronectin and laminin within the extracellular framework have contributions to tissue remodeling. Fibronectin mediates cellular attachment and function, and it has been implicated with tenascin in the promotion of fibroblast migration during embryogenesis and wound healing^24–26^. Furthermore, components such as laminins are found in the basal lamina and regulate cellular differentiation, proliferation, and migration^27^. Although these components are integral in understanding endothelial injury and cellular differentiation, the emphasis of this manuscript was tailored specifically around the major fibrous proteins collagen and elastin. The mechanism by which a HF diet may contribute to aortic valve calcification and degeneration may be multifactorial. The decrease in tensile strength and elasticity is from an increase in collagen types 1 and 3 and loss of elastin in the HF diet group. Additionally, the initial stages of microcalcification were appreciated in the HF diet group with staining and imaging. The focus of the current study revolves around the observed architectural properties of the extracellular matrix (ECM) and the physical changes that resulted from a high fat diet.

### Role of Osteocalcin and Osteopontin in Aortic Calcification

Similar to atherosclerosis, the role of endothelial progenitor cells (EPCs) continue to emerge as a key component in aortic valve degeneration^28^. In patients with coronary atherosclerosis, Gossl *et al*. and Flammer *et al*. proposed that EPC mobilization from the bone marrow may explain a relationship between bone metabolism and vascular response to endothelial injury^29,30^. The observations found in the coronary vasculature have also been expanded to involve the aortic valve^31^. Gossl *et al*. recently reported that circulating EPC-OCN had a significant role in the pathogenesis and prognosis of severe calcific aortic stenosis^32^. In the same study, it was observed that patients diagnosed with severe calcific aortic stenosis had a combination of decreased total EPCs, but increased EPC-OCN percentage, which may have contributed to accelerated vascular and valvular calcification^32^. Chronic diseases, such as hypercholesterolemia, hypertension, and diabetes mellitus, have been linked to increased incidence of calcific aortic valve disease^33^. It was observed that in the HF diet group, an expression of osteoblast-derived proteins osteocalcin (OCN) and osteopontin (OPN) were concentrated along the aortic side of the valve cusp. These findings suggest that (1) proteins present in ectopic calcification of the coronary arteries are also present on the aortic cusp, (2) tissue calcification may be specific to the fibrosa and ventricularis layers, and (3) MetS facilitates osteoblast-derived protein mobilization (Fig. 7). Similar studies have shown OPN localization within the aortic fibrosa further promoting the theory of layer susceptibility^34–36^. The current study shows that hypertension was observed in the HF diet group. It may be speculated that this increase in hemodynamic pressure from vasculature narrowing compounded injury to the weakening aortic cusp endothelium. Endothelial injury interrupts ECM composition and alters the communication between valvular endothelial and interstitial cells that are integral in determining cusp functionality and response to blood flow^36,37^. Sider *et al*. showed that calcific aortic valve disease results from valvular ECM disruption leading to valve degeneration and osteogenic metabolism^35^.

### Role of Lipid Accumulation in Smooth Muscle Mineralization and Degeneration

Several studies have suggested the role hyperlipidemia has in the mechanism of aortic valve degeneration^14,38^. Circulating lipids accumulate within the subendothelium and deposit on the aortic and ventricular sides of the valve. In Fig. 7, the enzyme lipoprotein-associated phospholipase A_2_ (Lp-PLA_2_) attaches to the substrate oxidized low density lipoproteins (oxLDL) to produce the proinflammatory lipid mediators free fatty acid (FFA) and lysophosphotidylcholine (LPC)^39–41^. In human clinical studies, Mahmut *et al*. observed varying expressions of Lp-PLA_2_ with respect to the severity of aortic stenosis. Findings from these studies suggest that lipid metabolism of LPC and FFA may promote vascular smooth muscle cell mineralization and activation of the tissue degradation process (Fig. 7)^3,4,41^. In the current study, increased Lp-PLA_2_ expression was similarly observed in specimens from the HF diet group. It is important to note that the HF diet group had a significantly increased lipid profile when compared to baseline. On histopathology and western blot quantification, an increased expression of Lp-PLA_2_, also referred to as platelet activating factor acetylhydrolase (PAFAH), was observed in the HF diet aortic cusps. Mahmut *et al*. showed that Lp-PLA_2_ greatly contributes to valve mineralization by the production of LPC, and the plasma level of oxLDL is directly associated with stenotic aortic valve remodeling^3^. The current findings promote that the experimental MetS model is conducive to studying the effects of lipid metabolism on the aortic valve. It may be of further interest to investigate the role of Lp-PLA_2_ inhibition to ameliorate the progression of tissue degradation^3,39^.

### Communication Between Valvular Endothelial and Interstitial Cells Lead to Fibrosis

The aortic cusp consists of valvular endothelial cells (VECs) and valvular interstitial cells (VICs) that communicate with each other to determine ECM functionality. Valvular interstitial cells differentiate from quiescent VICs (qVICs) into activated myofibroblast-like VICs (aVICs) and/or osteoblast-like VICs (oVICs) that are responsible for ECM remodeling and calcification, respectively (Fig. 7)^42,43^. Although VIC phenotypes have been extensively studied, differentiation of these cells are not completely understood^44^. In the HF diet group, the myofibroblast-like phenotype was appreciated with alpha-smooth muscle actin (α-SMA) expression. There was a significant increase in collagen deposition and active remodeling, but loss of elastin that altered the aortic cusp mechanical properties. Despite the role collagen plays to maintain cusp stiffness, pathological remodeling from activated myofibroblast-like cells may explain why cusps were weaker and less elastic. The predominant cellular component found in the aortic valve ECM are collagen types I and III^45^. Eriksen *et al*. observed that calcified valves have increased collagen type I synthesis with a slight upregulation of collagen type III^46^. While Rodriguez *et al*. observed aortic valve collagen decreases with age; the current study shows an increase in collagen types I and III in the HF diet group^45^. Findings from mechanical testing further confirmed that when compared to the normal diet group, cusps from the HF diet group were less elastic due to loss of structural uniformity in the ECM. On average, cusps from the HF diet group were thicker compared to the normal diet group, which may be attributed to fibrotic change. Although calcification was present in the fibrosa and ventricularis layers, there was also an increase of collagen deposition in the fibrosa and spongiosa (Fig. 3). These findings show a possible connection between the mechanisms of collagen deposition alongside calcification specific to the fibrosa layer. Further investigation regarding this link should be evaluated in future studies. Continuous activation of VICs may lead to increased valve cusp fibrosis and decreased valvular mechanical flexibility^47–49^. Despite different cell responsibilities, studies indicate that there might be an additional mechanistic step involving myofibroblast-like cells that leads to the initial stages of aortic calcification^43^.

The findings observed in this study have numerous implications in regards to VHD. For the first time, the effects of an experimental MetS pig model show that in addition to atherosclerosis, aortic calcification and degeneration may be related to a HF diet. The current study extends these observations and suggests a potential mechanism and therapeutic target for aortic valve degeneration in a high-risk population^5,36^. Other studies have investigated similar pathways and proposed therapeutic targets that would mitigate the effects of MetS and associated comorbidities^50,51^.

## Limitations

In future studies, it is important to explore the effect of therapy and reversibility of factors associated with a HF diet. The duration of diet given in each group has been observed to be sufficient to study the effects of MetS; however, allowing the study to take place for a longer duration may induce more of the degenerative effects of HF diet paralleled with age. In addition to micro CT analysis, a high resolution non-contrast CT with calcium score may be beneficial to image the aortic root, providing better insight into the degree of aortic calcification present. Additionally, measuring the aortic cusp size and weight would help further assist in determining the physical properties of the aortic valves. Biomechanical forces exerted in valve cusps may be equal in both the circumferential and radial directions of the cusp. Variability of the cusps is based on the morphology and structural composition of the valve. The aortic valve cusps experience a complex bidirectional flexure. Since the circumferential direction is the major curvature in the cusps, uniaxial mechanical testing focused bending in that direction^6^.

## Conclusions

In summary, the current study demonstrated that experimental metabolic syndrome is associated with mechanical and structural changes of the aortic valve leading to degeneration and early calcification. The pathological processes involved in the initial stages of aortic calcification are similar to the well-known pathological processes involved in atherosclerosis. Additionally, a HF diet was postulated to cause VIC differentiation leading to further expression of osteoblast-like factors and fibrosis of the aortic cusps. Clinical vigilance in improving these problems and understanding the mechanisms associated with VHD is essential to overall cardiovascular health. In the past decade, the main focus has been atherosclerotic risk factor assessment and modification. Currently, there are still no preventive medical approaches to early detection and attenuation of the progression of aortic valve calcification and degeneration. While surgical intervention remains the gold standard of therapy, findings from the current study suggest that an approach similar to atherosclerosis should be explored for aortic valve disease.
